# Religion and public health: conceptualization and collaboration from a public health perspective

**DOI:** 10.1186/s12889-026-26411-7

**Published:** 2026-01-29

**Authors:** Anish K. Arora, Johnathon Ehsani, Beth Resnick, Yovania Dechtiar, Andrew Hatala

**Affiliations:** 1 Department of Health Sciences, Wilfrid Laurier University, Waterloo, Canada; 2 Johns Hopkins Bloomberg School of Public Health, Baltimore, USA; 3 College of Community and Global Health, University of Manitoba, 221 Human Ecology Bldg. 35 Chancellor’s Circle Winnipeg, Winnipeg, MB R3T 2N2 Canada

**Keywords:** Religion, Public health, Collaboration, Population health, Wellbeing, Community health

## Abstract

**Background:**

Religion and public health, when understood in their broadest sense, are systems of knowledge and practice that seek to improve the wellbeing of people and populations. With over 75.8% of the global population identifying with some form of religious belief, understanding how the public health community conceptualizes religion and engages with it is essential for meaningful collaboration. The purpose of this study is to explore the conceptualizations of and relationships between religion and public health, focusing on public health practitioners’ experiences in engaging with religious communities.

**Methods:**

This qualitative study involved interviews with 15 North American public health practitioners across diverse religious and ethnic backgrounds, examining their perspectives on religion’s role in health interventions, with a particular focus on the COVID-19 pandemic.

**Results:**

Using a framework analysis, three key thematic areas emerged, including: (1) Conceptualizations of religion and how it contributes to individual and collective wellbeing; (2) Current relationship between public health and religion; and (3) Opportunities for and strategies to establish deeper collaboration. The results reveal a strong perception among public health professionals that religious communities are being engaged in an instrumental and transactional manner to achieve public health’s agenda, rather than as true partners that co-design health interventions. While some religious communities are compliant with such interactions, many are reluctant in engaging with public health communities. To move away from instrumental and transactional interactions to meaningful engagement, interviewees share numerous strategies consistent with the principles of community-engaged research (e.g., engaging with religious communities with sincerity, humility, transparency, and valuing the knowledge these communities possess).

**Discussion:**

The findings suggest that the prerequisites for effective engagement between public health and religious communities is based on foundational principles such as trust, transparency, and the need to identify points of commonality in order to organically work towards improving the health and wellbeing of communities. Putting these principles into practice raises questions about concepts such as expertise, access to knowledge, problem identification, and prioritization. In addition, it challenges the current structures and financing mechanisms for public health practice. Integration of religion into public health education could serve as a foundation to foster more inclusive and integrative public health practice. While more research must be conducted with broader samples, insights from this study underscore the potential for more inclusive public health approaches that better address the diverse spiritual and cultural landscapes of global populations.

**Supplementary Information:**

The online version contains supplementary material available at 10.1186/s12889-026-26411-7.

## Introduction

Over 75.8% of the global population identifies with some form of religious belief [1]. Even as affiliation with formal religious communities has steadily declined, religion persists as a strong social force [2]. Religion can shape individuals’ values, behaviors, and community structures, all of which can have profound implications for public health outcomes [3, 4]. Individual religious beliefs also influence how people perceive health interventions, adhere to medical advice, and their behavior vis-a-vis health prevention-related recommendations [5–9]. In many global contexts, religious institutions serve as a platform for health promotion, especially in areas where governmental or healthcare infrastructure is limited [10–12]. Together, these ideas suggests that the field of public health could benefit from advancing knowledge on how religion interacts with public health both at an individual and collective level [4].

Among the primary purposes of public health as a system of knowledge and practice is to secure and protect the health of people and populations. Similarly, religion, which in the broadest sense can also be understood as a body of knowledge and practice, also has a role in the promotion of societal health and well-being [13–16]. Indeed, Idler and colleagues argue that the overall goals of religious institutions and public health are shared, in that both “strive to improve the quality of life” and that they are both “mission driven” [17]. Given that both public health and religion share a common aim, and that both public health and religion have implications on human behavior and community life, the degree to which these two systems of knowledge and practice can deepen collaboration could be beneficial for public health agencies and practitioners [17, 18].

Yet, engagement across public health and religious communities has often been a challenge [18], particularly during public health emergencies such as the COVID-19 pandemic. From the public health lens, at best, engagement with religion has been instrumental or reductionist, seeking endorsement of interventions and treatments [19, 20]. At worst, divergent views have resulted in direct conflict and division [21]. There is a critical need, therefore, to learn how to create authentic dialogue between public health and religious organizations to advance the common goal of creating healthy and flourishing communities [17]. In order to advance this understanding, in this study we explored how public health practitioners defined both religion and public health, what their experiences with engaging religious communities has been across various public health interventions, and how they perceive engagement could be improved. By examining their experiences, this research uncovers some practical challenges and opportunities faced by public health practitioners when engaging with religious communities in designing and disseminating health interventions.

## Methods and approach

### Study design & participants

A qualitative descriptive study was conducted to explore how key informants define religion and public health, and how they perceive and experience interactions between public health and religious communities. This report presents a qualitative analysis and follows the consolidated criteria for reporting qualitative research (COREQ) guidelines [22] (see Appendix 1 in supplementary files). Leaders in public health academic settings working in the state of Maryland were initially recruited to take part in this study. Initial participants were recruited through email. Snowball sampling was then used to identify and recruit new participants from across the United States that come from a range of religious and spiritual backgrounds and have experience in working with religious communities for public health interventions either across Maryland, the United States, and/or internationally. A total of 15 key informant interviews were conducted. Participants included: a public health chief, institutional directors, professors, physicians, a political scientist, a health economist, senior decision makers, and health advisors. Interviewees also ranged in their ethnic backgrounds (i.e., a mix of people from Asian, African, and European descent participated), as well as their religious affiliations (e.g., Baha’i, Buddhist, Confucianist, Jewish, various denominations of Christianity, etc.). No participants refused to participate or dropped out. No prior relationship between research team members and participants were previously established prior to study commencement.

### The research team

The research team consists of 4 PhD trained researchers and 1 MPH trained researcher. All team members had prior experience with qualitative research, with the first and last authors having expertise. The research team also included 3 males and 2 females. At the time of this study, 3 team members were associate or full professors, 1 was a postdoctoral fellow, and 1 was a research assistant. The religious orientations of the study team vary. The team came together through an international reading group facilitated by the first and last author through the Association for Baha’i Studies of North America to explore the relationship between public health, science, religion, and spirituality.

### Data collection & analysis

All members of the research team participated in developing an interview guide over the course of three meetings (see Appendix 2 in supplementary files). One-hour-long semi-structured interviews were conducted via Zoom videoconferencing software. Interviews were led by the second author (JE), with support from the rest of the research team. Only the participant and research team were present during the virtual interviews. All interviews were audio-recorded and transcribed verbatim. Field notes were taken during the interviews to provide contextual information for subsequent analysis. No repeat interviews were conducted and participants were not provided with transcripts for comment or correction. Transcripts were entered into a qualitative data software (Dedoose coding software Version 9.2.11) [23] which allows text to be coded and retrieved for ease of summarization and interpretation.

Qualitative data analysis was conducted using the framework analysis method [24, 25], where codes and categories were generated after each interview. Two investigators (AKA and AH) coded all the transcribed interviews independently. Regular virtual meetings were conducted among authors to compare codes, address any discrepancies, and facilitate the identification of emerging themes. Initially, a deductive approach was employed for coding the transcribed interviews, with codes derived from the framework outlined in the interview guide. This was followed by an inductive approach, allowing the categories, patterns, relationships, associations and themes to be generated directly from the transcribed interviews, leading to a broader thematic framework. Key themes were identified through an iterative process utilizing a constant comparison method [24]. The final analyses were reviewed by the entire research team ensuring multiple perspectives were considered, deepening the understanding of the data, ensuring internal quality control and rigor, and supporting thematic completeness.

### Ethics

Research ethics approval was received from the Johns Hopkins School of Public Health Institutional Review Board (Number: IRB00025023) and consent was obtained from those participating in the study.

## Results

Based on the perspectives of participants, themes that emerged from the interviews were characterized according to three areas: (1) conceptualization of religion and how it contributes to individual and collective wellbeing; (2) current relationship between public health and religion; and (3) opportunities for and strategies to establish deeper collaboration. Across all three areas, multiple viewpoints were expressed, often in contrast, reflecting a diversity in the perceptions of the public health community. For each of the three themes, a table with further detail is included. The tables highlight all categories, sub-categories, and representative excerpts (include examples from interviewees) for each theme.

1. *Conceptualization of religion and how it contributes to individual and collective wellbeing*

Almost all participants began their interviews by sharing their personal experience with religion. Several expressed having been raised in a particular religious tradition and how their academic pursuits often pushed them to challenge fundamental assumptions of their religion and/or their beliefs. Increased interaction with people from other religious communities and ethnicities, as well as a deeper dive into scientific theories and principles often pushed many away from organized religious practice. Here, some also cited the fact that religion has been used by leaders as a means of disunity and harm, which further dampened their appreciation of institutionalized religion. Several mentioned that when they moved away from institutionalized religion, they felt they were able to build a closer relationship with “a divine source” – an act which many referred to as practicing spirituality.

Interestingly, many interviewees expressed re-connecting with their religious traditions over time or exploring new religions, particularly during the COVID-19 pandemic. Despite recalling the harms that have been carried out in the name of religion, participants overwhelmingly described how religion can be a source of many benefits to individuals and societies. Interviewees expressed that, in their perspectives, religion ultimately provides people with a moral framework, a guide for life, purpose and meaning, and a philosophical understanding around “spiritual principles and reality” that in turn helps people collectively navigate what is good and bad, raise global consciousness, provide the groundwork to establish true global peace, unity, and happiness, and facilitate individual and collective flourishing. Participants also noted that membership within religious groups also affords people with access to social networks and support systems, which can be important for individual and community well-being.

When asked to further define religion, almost all expressed this as being a difficult task. Rather than defining it in its entirety, alongside the discussion around the purpose of religion, participants often broke religion into five other constituent parts: spirituality; traditions; institutions; jurisprudence; and historical context and narratives. Subsequently, when asked to define public health, participants were quick to offer concrete definitions, citing its focus on establishing and maintaining optimal physical, mental, social, and spiritual health across the life-course, often through preventative and upstream interventions centered at the population-level. Importantly, after expressing their definition of public health, some came to describe a realization that both religion and public health: offer opportunities and methods for safeguarding the health and wellbeing of people; are focused on serving humanity; and are aligned in their aspirations for social justice. See Table 1 for more details on this theme.

**Table 1:** Representative excerpts for theme 1 – “conceptualization of religion and how it contributes to individual and collective wellbeing”

Category	Representative Excerpts
Change in religious identity, affiliation, and/or practice over time	“I was born into a Christian family and who were practicing their faith. At the time they were Baptist. And so I was born into a Baptist family. However, they sent me to Catholic schools. And because of that, and having religion classes every day and having to go to math once a week, I eventually became Catholic … I then went to a Methodist college … And so then I began to chat, having questions and challenging just the different approaches. So I went off [to] practice Buddhism for a little bit, and circle back around to, which is akin to unity Christian Center … got married and landed back in a Baptist church. So I came full circle. But my way of practicing is really integrated with all of those approaches.” – Participant 2 “[As] a teenager, as I said, I was not interested in religion … By the time I was a 41 year old woman going through a divorce, right, like life, like had meat tenderized me, I was open to religion. Because I just realized after doing all the things that I [had] done, like I needed something that was bigger than me. And when somebody walked into my life with the Baha’i Writings, I got a vision of religion, especially because in part, I mean, the Writings were great, and they were powerful. But I think also what really grabbed me was the was oh, community building efforts and like that kind of learning culture.” – Participant 7
Religion as a source of disunity and harm	“So religion is an institution, right? So religion is critically important to, I would say everyone’s life, regardless of what your faith is, religion will impact you in some way, shape, or form, even if you’re an atheist. Because the things that divide us are politics and religion, and oftentimes religion influences politics.” – Participant 5 “I thought, like, religion was religion, it was the problem. The problem started with religion, and it ended with religion, because there was just nothing that I saw that indicated that religion had anything to offer problems that people were dealing with. It just did not.” – Participant 7
Religion vs. spirituality	“So I am a Christian, so, and Baptist denomination, my relationship with religion, I guess kind of ebbs and flows through the traditional lens, I consider myself to be probably more spiritual than religious. There have been times in my life where we grew up in church and church was a huge focal point. And there were other times where it was not, but I still always had a personal relationship with God and still, you know, still prayed, still, you know, worship in my own way, we just may not be in the house of the Lord, per se.” – Participant 5 “Okay, so I think religion and spirituality are two different things. I think religion is religion is very, it’s very defined, in every religion has its tenets that people are supposed to abide by… Spirituality, I think is more about your relationship with God, if you believe in God, or the gods that you believe in, or just the idea that they’re, that we don’t live in this life alone, in that this life is not limited to what we see. Or what we feel there probably other planes in this this in this world and how you connect, and how you connect to that.” – Participant 6
Re-connecting with religion during the COVID-19 pandemic	“Yeah, so I’m Jewish and I would say in the last several years, I’ve become much more spiritual … so I do Torah study, I do a lot of reading and things. I’m not observant in the sense that I don’t go to services every week or keep Shabbat in the sense, but I’m very intellectually curious about religion. And I think that got accelerated during COVID, when I saw so many things happening in the world … and the political divisions and things. And I found it very helpful and comforting to look back, particularly when you start reading the Bible, and you realize that all of these things happened 5000 years ago … It’s so similar today, it’s kind of almost eerie. So I have been finding that very helpful.” – Participant 8 “I don’t consider myself a practicing Catholic in the same way I was when I was younger, and in the family context. But I would say in the last few years, I have tried to. I’m coming at it more from a spiritual angle, and have noticed that. And it became even more so during the pandemic, and I had to do some, some [thinking] about whether my soul was being fulfilled. And I could just be more present in the world and present for people in a way that was not instrumental. But personally, I have not until recently nurtured that aspect since I was a kid.” – Participant 10
Defining Religion - Purpose o Moral framework o Guide for life o Purpose and meaning o Philosophical understanding around spiritual principles and spiritual reality o Individual and collective consciousness o Peace, unity, and happiness o Human advancement and flourishing o Building communities and establishing/maintain strong social ties - Constituent parts o Spirituality o Traditions o Institutions o Jurisprudence o Historical context and narratives	“For me, I think religion is what grounds the individual, it is the ethos by which they are who they are. Religion is … the thing which is your conscience by which you operate. Religion is how, for me, how I see the world, how I experience the world and how I see myself supporting individuals in the world … And I recognize that based on my religious beliefs, that I am here with a purpose … and to give back in ways that only I was created to do, and not in a conceited kind of way, but in a way that recognizes that we are all born with a gift. And our gifts are to be used to be of support and to be of help to others … so, sort of religion and spirituality in the broadest sense … is a meaning making enterprise. I don’t want to use the word enterprise, but it, you know, it fulfills this human need to make meaning and to make social connection.” – Participant 1 “So when I think about the definition of religion, it goes to that the purpose of religion is to really contribute to peaceful coexistence of humankind, you know, and to really foster a truly global consciousness and restore the bonds, you know, between different people, too. So, unfortunately, when we think about how to define religion, people usually get stuck in the beliefs of that particular religion, or the tenets or commandments, or who they worship. And you know sadly, religion has played a role to divide people, and there have been so many conflicts and wars in the name of religion. But if we really think about religion, it’s a source that can unify people. It can. It can bring people together; it can contribute to peaceful coexistence. So for me, religion is like this driving force that can help me to polish my own heart, my own mind, elevate my own global consciousness, live a life of deeper purpose and meaning while really treasuring each person and each encounter. I have to really solidify the relationships among people, both in my own immediate life, and then what I can do to bridge the gap between people and nations beyond me.” – Participant 15
Defining public health - Establishing and maintaining optimal health across life-course - Generally preventative and upstream - Also responsive to immediate and emergent issues - Responsible for physical, social, mental, and spiritual health - Transdisciplinary field of inquiry and action requiring multisectoral collaboration - Population vs. person-centered lens	“Well, public health is in a real sense an acknowledgment that there are ways in which we can organize our society such that our populations can maintain and not just maintain health but achieve good health … Essentially, this fact that whether we’re talking about a physical environment, built environment, the health care system, food environment, you know, all the contexts in which we live and breathe and move in, that if we don’t construct them appropriately, then we’re going to suffer code collectively. … Public health helps society make decisions around how to order itself, such that we all sort of benefit from a good environment, places. And so think about, even, you know, we’re in this this season of, of natural disasters. And, in part, some of those disasters can be anticipated, and you can mitigate the risk of harm associated. And so even when you think about things like building design …. you can make a lot of money [building places] doing it that way. But you also put a lot of people at risk, right? And all we need is a few tremors in your building.” – Participant 3 “So I think of or I would define public health as both a study and a practice. But it’s the study of identifying those factors that either contribute to, or cause, or may address, population wide patterns of health or conditions, both sort of chronic conditions or infectious conditions, but it’s the study of those factors that cause or contribute to population [health]. I’m thinking of sort of patterns, disparities, discrepancies, prevalence of conditions. That, and then, the practice part is trying to identify interventions that can help address both disparities, but also sort of prevalence even when you don’t have these really clear populations that have higher or lower rates than other populations.” – Participant 14
Recognition of the shared aims, purpose, and goals of religion and public health - Both offer methods and thinking around safeguarding the health and wellbeing of people - Both focus on serving humanity - Both are aligned in their aspirations for social justice	“We were talking to residents in Zika affected communities in the United States … But this is the people’s explanations about their experience with Zika, or their willingness to, for instance, be okay, with aerial spraying to kill adult mosquitoes. They gave very spiritually framed answers as to why they thought and did. And it just, it kind of hit me, you know, and it’s like, wow. And so there was one woman who, who’s like, ‘you know, I hate the idea of chemicals in the air. But I’d be willing to do it because we have to worry about the body of Christ [which] includes everyone. And I have to worry about the last, the least in the last, and so there are landscapers who are outside all day or construction workers, so we need to protect them. There are people who can’t afford, you know, products over the counter products to keep mosquitoes away, like *off*.’ And so I thought, wow, that is so powerful. I mean, that’s how she thought about it. That’s how she sort of tamped down any qualms she might have about her own needs and elevated the needs of others around her, and she used a religious framing to do that. And I thought, wow, wow. Now, especially in the US, because we have this problem with church and state separation lately, I wouldn’t want to see every health officer in the United States, you know, reframing everything that public health does from that perspective, because it’s exclusive of people or it can be exclusive of people. At the same time, there is power here in that. These ideas of wellbeing and collective, well, collective wellbeing and responsibility to others. It’s like, that is just pure public health.” – Participant 9 “I feel health is not just absence of illness or sickness, but health is this ability to live life that is true to oneself, and filled with courage, filled with hope, you know, filled with a sense of direction, making a positive difference in our day-to-day life, and you know life beyond our own self. And that’s why I feel like, you know, when we engage in [public health] or when we engage in spirituality … it all promotes health.” – Participant 15

2. *Current relationship between public health and religion*

Though some were able to identify shared aims and purposes between religion and public health, almost all expressed that, practically speaking, the relationship between these two communities at present was primarily instrumental. This dynamic was characterized by public health agencies and practitioners primarily seeing religious communities as a means of accessing a population for the purposes of disseminating a health program or information, such as health education delivered at the time of a religious service, or health screening conducted at a religious facility in collaboration with a local community.

Although several interviewees stated that their congregations welcomed and respected the expertise of the public health community, many described disconnect between the goals of public health and religious communities. For example, “*religion doesn’t see public health*” and “*there is no relationship between religion and public health*” were expressed by several individuals. One interviewee described an anecdote where their religious community organized a large gathering immediately prior to the implementation of COVID-19 distancing measures, knowing that the opportunity to gather again soon may be unlikely. This led to the transmission of the disease within their community. In this instance, the religious community’s goal of maintaining the cohesion of the congregation was in direct conflict with public health goals of social distancing. Similarly, another interviewee expressed that public health and certain religious communities can be in direct conflict with respect to certain issues, such as women’s reproductive health.

While many participants were able to describe instances of problematic engagement between religious and public health communities, most were also able to share some positive examples of productive engagement between the two groups. Participants shared that in certain contexts, where strong partnerships existed, public health professionals were able to campaign and provide many services (e.g., cancer screening, awareness and prevention education, blood donation drives) for months at a time. Some also described that genuine heart-felt collaboration between religious and public health communities arose not because of either group’s initiation, but rather because external forces motivated communities to build stronger ties of cohesion to combat racism.

However, when contemplating why respectful and effective engagement between the two communities can be difficult, participants expressed numerous reasons. Participants explained that people on both sides of the community often think that religion and science are antithetical; that if one believes in God, they cannot believe in science, and vice-versa. Further, participants described that religious communities, in particular, do not trust or feel comfortable engaging in conversations with public health professionals. This lack of trust and discomfort is amplified by historical challenges, particularly with racialized and marginalized communities. Some stated that a lack of opportunities and spaces to engage in open and safe dialogue prevent genuine collaboration, and further, that people from both religious and public health communities may not always understand what the other group actually knows, how they think, what they do, and how they work towards improving the health and wellbeing of others. In this regard, some participants expressed that many may not question their own assumptions and biases before engaging in discussions with the other community.

Two participants also discussed experiencing hypocrisy, one of which expressed the dire concern of people from religious communities sending death threats to public health experts. The other, expressed that people from public health communities engage in practices and endorse work that is not meaningfully engaging religious communities or necessarily responding to their needs. Concurrently, several participants described situations in which one group forced their belief and/or mandate on the other, for example, with public health shutting down all voices that were against vaccination during the pandemic, or with religious groups trying to prevent abortions in certain jurisdictions. A few participants also shared that public health and religion are “big businesses” that influence and are influenced by politics. This necessarily creates divisions, and can hinder people from meaningfully engaging with one another, for example, when public health professionals must place their accountability with funders rather than the religious communities. See Table 2 for more details on this theme.

**Table 2:** Representative excerpts for theme 2 – *“current relationship between public health and religion”*

Category	Representative Excerpts
Current relationship between religion and public health is primarily instrumental	“I think research organizations and academic institutions and healthcare systems see the faith based organizations as opportunity, as sectors of individuals and communities that can be reached, and are in specific spaces. I think it’s, it’s probably easy to say, ‘oh, let’s talk to this faith based organization’, because it’s easy to identify who those faith base either congregations or churches or temples are, and synagogues. And to be able to say, we’re going to this faith based institution to do X, Y, and Z. And knowing that [these] organizations have a foundation for how they are engaging with their congregants. And so it might be easy for researchers or like institution to be able to say, we’re going to use the institute, the faith based institutions as places to do good research or lots of research or to gather data or to get input. I think it’s, I think academia, values, community, faith based communities. I think that they’re also easy, easy in terms of, they’re easy to identify.” – Participant 1 “So a lot of public health work, or some work, says, engage with faith based communities because when you’re thinking about who are your stakeholders, who are your gatekeepers of infamy, who are the gatekeepers of communities, you need to think about who those people are, and faith based leaders are always going to be on that list. And again, it’s irrespective of your religion and your denomination, etc. Because religion is such an important part for most people’s lives. I don’t know the stats, but I’m pretty sure most people are connected to a religious faith in some way, shape, or form. And so there needs to be their intersection of public health and religion, because you need to design programs that are going to bring people to the program. So it’s not a built in, they will come mentality, it’s a let’s tailor a program to meet their needs. So let’s say, I don’t know heart disease prevention, as an example. So if you’re designing a community based program, then you definitely want to bring that in those faith leaders. So you know, you can get them engaged, get their buy in and support and then be able to deliver that same curriculum to their congregation, for example. … And so when you have those faith leaders who are trusted leaders in those communities, you can tap into their network and leverage that for them to help cross promote healthier messages because people follow two people blindly: their doctor and their pastor. So when those two people say something, they are going to listen and most times listen without pause, listen without questioning, they’re going to blindly follow those two people because particularly in a black community, in my personal experience, that’s what we’ve been taught that these two are the people who are the authorities that we need to pay attention to. Right? So it’s very important that as public health practitioners, we tap into that faith based community and we don’t ignore it. Because it is it’s viable, it’s robust. And we can help expand our programs expand our reach, by tapping into the faith based community. – Participant 5
Many instances of problematic engagement	“I think what frustrates public health experts is that when the information that they have to share is not appreciated, [people don’t] listen to [public health] folks, go ahead and do things, and then instead of blaming themselves for not listening, they attribute their fate to God and say, you know, obviously this happened because it happened, right? And it’s like, yes, no, God doesn’t want you to get in a car accident and that’s why there are stop signs, spiral traffic lights, and if you ignore them, then you’re gonna end, it will not be that God is looking for you to come and because [you] didn’t pay attention to the traffic light.” – Participant 3 “So in my experience, I think that the parts of the public health community that sort of, that are, that I’m most aware of, they regard religion very negatively. They regard religion as antithetical to really protecting and respecting health and life. And I think that’s largely because there has been, there have been, some faith based organizations that have played an outsized role in trying to shut down access to contraceptives or abortion or put into place, the most limited and almost inoperable exceptions for when these otherwise banned services are available. Similarly, the LGBTQ policies work. The sort of quintessential Florida ‘don’t say gay laws are the bans on gender affirming care for minors.’ There have been a handful of very effective and very well funded faith based organizations that have really played an outsized role in trying to shut down, sometimes target, and very much I think, hurt, some of the community. So I would say that in my day, based on the areas that I’m most interested in, I’d say people who work in that field of public health would regard religion as a very negative or potentially harmful actor in that field of public health. … In the areas in which I’ve worked, it seems to me that faith based organizations would view public health negatively, or the goals of public health negatively. … The objection to vaccines even within my faith, there’s an Orthodox Jewish movement that’s really against vaccination. And there is no religious based reason for this. In fact, in Judaism, you’re supposed to, your health prevails over any other competing interests. But I’ve seen sort of distrust and hostility by some faith based organizations or leaders toward public health, the, you know, the limits on the number of people who could gather for worship. So I would say, and this is based on my work, so I know that it’s not generalizable. But I would say I’ve seen some distrust or hostility by some religious advocates toward public health and the kind of mission of public health. – Participant 14
Examples of positive engagement	“There is the red dress Sundays in February for Heart Health Month. So churches all across the US, right, everyone is wearing red, and there are mobile units outside where they’re doing blood pressure checks, and there’s lots of different hospital and university systems who are literally on site, like during that Sunday service, or that Saturday service, and they are providing screenings to anyone who was walking in, and it doesn’t matter, like what organization they’re from, or it’s, you know, a blood donor drive, and, like, folks are there and they are willing to engage, and they’re willing to have conversations, and they’re willing to do checks and look at the different types of medication. … And so I think, I think those examples like really work where it’s intentional, where there are groups that are set up in advance for like, the entire month, because this is what we’re doing in the month of February, or it’s, it’s October for breast cancer awareness. And you have like, organizations who are there, again, with mobile units, talking about breast self exams, and why it’s important, or during October for Prostate Cancer Awareness Month … And again, this is this, I can speak for the black church, because I know that that’s happening. …COVID was another great example, it was like, you have individuals who are talking at the minister level, and the pastoral levels, talking to their congregation about the importance of getting your COVID vaccine, the importance of getting flu shots. And so while public health officials may not be able to get that message across, or be able to, to have that translated in a way that the community feels they can trust, when you’re hearing that from your faith leader, you are more inclined to go ahead and do those things, because you’re hearing it from a trusted member who is part of your religious community. And I think those things work really, really well. And I know that I mean, I think for the Biden administration was very clear, and even the Trump administration was very clear about using faith leaders to promote some of this.” – Participant 1 “I never saw a partnership between the Muslim organizations, Islamic organizations, and the Latino, Latin X, community. Never. … In 2016, we saw the creation of a movement called tacos, taco trucks at every mosque. And this was an effort to counteract the fear that some of the former president’s allies were stating in the news … So these two [communities] got together and said, well, guess what, we’re going to put taco trucks at every mosque, and it was around the time of Ramadan. … And still to this day, it continues. And the leaders of those inter movement spaces said, the reason why we’re doing this is because unless we sit together, and eat, share a meal, talk to each other, pray together, [we] will not solve this crisis; that this is not something we [can] do in separation. We do this only with solidarity.” – Participant 9
Challenges that hinder respectful and effective engagement - Consider religion and science to be antithetical - Lack of comfort and trust - Lack of opportunities to engage in dialogue - Lack of understanding around what each community knows, does, and brings to the table - Hypocrisy - Forcing one group’s belief and/or mandate on the other - Historical challenges - Business of public health and religion o For public health professionals: accountability to funders, not communities - Influence of politics	“I was invited to a think tank for the black Mental Health Alliance … And they asked [the panelists], specific question … ‘how do we collaborate with people outside of you know, our [organizations]?’ And I talked about, ‘don’t allow us to come to you with a prescriptive way of doing some something, tell us what you need.’ And this woman said, ‘Okay, I’m just gonna say this, because no one else is, we don’t trust you. We do not trust you. Right.’ So are you thinking about my colleague who is Caucasian, and she may decide that she now wants to work with pastors and churches, and you just go and try and call them up? It doesn’t. So you haven’t done your work or history to try and research, to try and understand, how to engage the people that we want to help and they’re not looking at it as being very helpful if you’re approaching [them] in an incorrect manner.” – Participant 2 “I know that there is, as you know, also some mistrust, distrust, miscommunication, misunderstanding. You know, there are certainly, in public health specialists, because there’s some folks who think that science and faith are incongruent with one another, and they can’t understand scientists or even scientific methods, and still be a man of faith. And so while they respect the institutions as trusted voices, they sort of say, well, you know, but they’re misguided voices, because we know better, or [are] smarter than that.” – Participant 3 “I am not, you know, some health guru like Dr. Anthony Fauci at the federal level, I’m not that person. I am a small professor in a small community that works with students predominantly in a university that caters to first generation students that are usually non traditional students. I’m not famous by any stretch of the imagination. I’m quoted in like local papers. But I got death threats. How is that possible that I’m getting death threats by the same people that go to church on Sundays, that claim to be religious and believe in God and yet harm the very people that are trying so hard to protect them in this society?” – Participant 9

3. *Opportunities for and strategies to establish deeper collaboration*

Interviewees expressed a range of viewpoints about opportunities for deeper collaboration. Though some interviewees described a common “instrumental” relationship as the status quo, and did not articulate a need or vision to move beyond this approach, many expressed the need for more responsible and authentic integration, partnership, and collaboration.

Interviewees noted that one primary approach to establishing better collaboration was through creating opportunities for and engaging in safe dialogue. In such dialogical settings, interviewees expressed the importance of focusing on finding common ground, engaging in meaningful discussions that seek to understand and respond to the needs of each member, and using both self-reflection and group-consultation. With respect to the latter, participants expressed how reflection and consultation enable groups to begin recognizing the value each brings to human wellbeing and flourishing. Personal and organizational reflection was noted as key to exploring assumptions and biases, and consultation was noted to help foster understanding of the other. Indeed, one participant discussed the vital need to provide more formal and informal educational opportunities about each group’s beliefs, perspectives, and ability to contribute to health and wellbeing, for each group to better understand and come to know each other.

Whenever interviewees shared the need for increased collaboration between the two groups, they often mentioned that true and meaningful engagement requires both parties to engage in a relationship building process, which they noted to take time and patience. Participants also expressed that this relationship building process requires recognition from both groups that there is a shared responsibility for making this work; that the burden of making the relationship work does not fall in only one group’s purview. Further, interviewees mentioned the importance of being authentic to build trust and solidarity, and tied to this, interviewees discussed the importance of truly loving the community you serve as a foundational tie that connects both groups.

Alongside loving one’s community, participants focused greatly on the importance of building and empowering communities as the central goal of both religious and public health communities. In this regard, some interviewees said there was a need to overcome saviorism mentality that many public health professionals often come to religious communities with, as well as the power asymmetry that exists between the two (i.e., when public health experts engage with lay community members).

When talking about community building and empowerment, interviewees also discussed the role of knowledge (i.e., expert versus non-expert) and the generation of knowledge more broadly. In most instances, public health researchers and practitioners described engaging with religious communities as experts delivering knowledge or programmatic content and that this is accepted or even welcomed by religious communities. Some interviewees stated that working more collaboratively with religious communities may provide insight into factors influencing health that are not traditionally included in programmatic work, including the importance of meaning, purpose, and the power of the human spirit. For religious communities, this would mean moving beyond being passive recipients of knowledge or services to being involved in the process of the generation of knowledge that advances community health. For example, rather than simply receiving knowledge about screening for diabetes, a religious community can be involved in building an intervention that supports their learning about metabolic diseases, helps them explore the history of their religious community and its practices around health and wellbeing, and ultimately, co-design an approach to helping members sustainably improve their health and wellbeing This would require that public health practitioners democratize knowledge, focus their efforts on relevant contemporary challenges needed by the community, and embrace the expertise of all people. Participants also express that this would require that public health practitioners engage with religious communities with sincerity, humility, transparency, enthusiasm, and optimism, allowing the relationship to evolve beyond instrumental and transactional engagement, towards the development of genuine collaboration built on trust.

Some participants also expressed that, for public health professionals to build comfort with engaging religious and spiritual communities, they must first be comfortable in kindly and openly discussing “the divine”, embracing faith-based language (e.g., being able to reference religious texts when discussing the importance of certain health-related practices and prevention strategies), and embracing complexity in measurement (e.g., recognizing that religious belief and practice cannot be captured by simple yes/no questions around belonging to a church, temple, synagogue, or other religious institution). However, while several interviewees described a desire to engage in such practices, they also shared a feeling of constraint by institutional structures that support and finance public health, which are based on short term funding mechanisms such as grants and one-off projects that do not allow for the cultivation of long-term relationships.

In instances where reciprocal comfort, trust, and meaningful engagement had been achieved, the interviewees described a synergistic and dynamic relationship between the public health and religious community. Overall, the importance of humility, recognition of the shared efforts towards building communities and improving communal wellbeing, as well as acceptance of different forms of knowledge, seem to be required by both religious and public health communities to ensure fruitful collaboration. See Table 3 for more details and participant quotes on this theme.

**Table 3:** Representative excerpts for theme 3 – *“opportunities for and strategies to Establish deeper collaboration”*

Category	Representative Excerpts
Though some do not question the status quo, many express the need for more responsible integration and collaboration	“I think in an ideal world, it would be a consistent and synergistic relationship. I think most times religion is an afterthought, or the faith based community is, is not considered one of the key stakeholders. But it’s a missed opportunity. Because the power of the religious community, first, there’s power in numbers, and the numbers are there, right? So that’s a built in audience. So if your pastor is telling you, you should go to this health event, your probably gonna take [it] a little serious, and think about it at least twice, before, you know saying no. So I think having, you know, faith based integrated into the work that we’re doing would be an ideal vision, because to me, they are a key stakeholder. And they have a very valuable perspective, particularly when we’re thinking about tailoring programs to different communities, right. So they have different insights and unique perspectives that could be valuable to how we go about doing our work. So that’s how I would see the vision for them to have a dedicated seat at the table, for us to work in a synergistic fashion towards the common good, because they want their own congregation to be healthy, just the way that we want them to be.” – Participant 5 “So I think there you have to find something that there’s common ground about, right, like that they, that the members all care about, and that you’re on your agenda, versus what I think we tend to do is come in, and here’s my agenda, at least in public health, I can’t say the other direction. And you know, we want to do X, and then the church might say, but we’re not interested, or the whatever religion it is, we’re not interested in X, we want to do Y, then unfortunately, again, you could peel back the onion. A lot of times it has to do with the funding source, but the public health agency might have or the goal or the you know, the criteria that they have is set on only x. And so if the group doesn’t want to do X, you’re kind of stuck. So, you know, so there’s a lot of reasons why these things might not work. But I think there needs to be a more specific goal and a mutual understanding of what you’re aiming to accomplish. Back to what’s a good partnership. And if you haven’t done that, then I agree then there what’s the value of it?” – Participant 8
Creating opportunities for and engaging in safe dialogue - Focusing discussions on finding common ground - Engaging in meaningful conversations - Use of reflection and consultation	“Honestly, the most important things are home visits, go visit your neighbors, connect at that individual level. I remember reading an article in the, I don’t remember where, but I was reading this article about how Halloween in the United States is the only time that we feel it’s okay to go and knock on the door of our neighbor and ask for something. … we need more of this. We need more. We need the ability and the courage, the moral courage, to go out and knock on someone’s door and say, ‘Hey, my name is …’ How many of us are mixing and not just on Sunday? Well, everybody separates in their own churches, but in spaces where we bring people together? … the only two things that will get us out of these health disasters are science and solidarity. … we got the science, you’re working on the solidarity. And that is, to me, that magic space. How do we create spaces, to bring groups together to have conversations one on one, meaningful conversations? I’m not just throwing words around like that, they have to be connected to hearts, right? And how do we then use that as an opportunity, perhaps, to share the importance of vaccines that gets like there’s spaces of trust, that maybe are getting more granular, that are getting more localized? And we know this from studies on trust, the more you know someone, the less likely it is that you won’t believe what they say. … And I think the parallel with the community building efforts is, [as] more of the community is engaged, you see, actually, the more impact on health and wellbeing vibrancy the community has. So it’s like you could see these parallels with community building, unity of vision, really understanding the complexity of the determinants of health and well being and how do we make sure that humanity can understand that, I think, science, the process of science, when I say science, I don’t mean mean it as a noun. I mean it as a verb, like the process of science and the scientific inquiry and methods that keep us open and keep us skeptical enough to ask questions, but not so skeptical that we become devoid of ethics and understanding that there’s a mystery we’ll never understand. … That secret of understanding this is in community. And we’re not focused there. We keep focusing on individuals and institutions, and we don’t know even how to unpack that community space, that space of integration, connection. And I think too often that science sits in this institutional space, so does religion or we look at the adherence as individuals. But what about that space, that belonging space, that community space? How is that being formed?” – Participant 9 “Were we there for each other? What do we do for each other? Were we there for our patients, clients, populations, congregants? Did we show up for them? And maybe some safe space conversations there, among leaders to just kind of explore this would be a good idea, you know? I don’t know, I could just see that it would have to begin with people talking to each other. And saying, you know, let’s explore this together. Whether it’s among leaders of these institutions, or down at these local levels, where it played out the local leaders.” – Participant 10
Recognizing the value each brings to human wellbeing - Taking time to explore personal assumptions and biases - Taking time to truly understand the perspectives, strengths, and limitations of each group - Potential need for increased educational opportunities to better understand each other	“The faith community realizes that they need science to some degree, right? They need it, like the people get sick. And the best outcome tends to come from the doctor, right? Like, that’s, at this point is undeniable. Right? Like, if you want to get well, the doctor usually has a lot they can do to help you. So you have to respect it. And at the same time, the public health community says, hey, well, there’s 100 people meeting at this church every Sunday. … And so there’s like a recognition that both people play important roles in the lives of the other. Visit your doctor or have this prostate exam, have this breast exam. And people do it right, like the whole congregation will do it. And that is very, very useful. – Participant 4 “I had also started thinking about the fact that, especially during the pandemic, that there was an impulse within public health to try and get above the fray of the politics by appealing to scientific knowledge, right, because it’s like, right, it’s supposed to be pure and objective and have everyone’s collective interests in mind objectively. And inadvertently, really started squelching views of others, views of the experience of the epidemic, and worldviews that directly affected people’s behaviors. During the pandemic, whether they were wearing masks, whether they were accepting of COVID-19 vaccines, and especially through the ethnographic work on vaccine access and acceptance, it’s like public health has to make room for other worldviews and ways of thinking … And for some populations, the scientific robustness really is the primary concern, and I don’t want to devalue that. But there are other worldviews that bring people to the same outcomes that a scientifically driven framework produces. And in fact, in some cases, are even more powerful than the science. And I think that there is a lot of value in public health really taking a pause and looking at religion and spirituality.” – Participant 10
Putting in time and effort to nurture relationships - Being authentic - Recognizing that relationships are bidirectional and, thus, both groups share the responsibility for making this work - Building trust and solidarity - Being patient - Loving the community you serve	“One of the times where I see it is often successful, is in relationship building, and having the understanding that the relationships take time to build, right, because you’re building trust, and it takes time. And it’s always about nurturing. It’s not about, you know, going to do something and then leaving. Right now, I’m still working with faith communities on a project that ended almost two years ago. And they still want to, I’m still supporting them and [helping them in] doing it themselves and embedding some stuff within the community, in the churches. They’re still doing it, I still support them. [I] fly out and go to one of their workshops, provide lunch, and then, you know, go back, but the relationship has to remain, you know, and it’s not nine to five, and so on. … This is my partnership with churches. And so in order to maintain that trust with the church, as the relationships take nurturing, and it always has to be. There is no, ‘I’m going to come to this and leave,’ it always has to be there…There is no shortcut for relationships …” – Participant 2 “Are you there for real and authentic reasons? Or are you just using somebody? Is this a manipulation? And so again, I think it is complicated, right? When there’s different beliefs. So abortion guns, maybe not even guns, but I would say abortion, for sure, is one where there’s a lot of strong opinions, or transgender care, or things like that, like those are where there could seriously be some strong disagreements, and both people have some valid points on their, you know, on their perspective. So, but again, I think about it with politics, right. Like you, you might partner with somebody on a piece of legislation, and then another piece of legislation you don’t agree on. So, but again, I think that has to do with the relationship. So I don’t think there’s a one answer. Does the church like the health department using them? Maybe in some communities? Yes. And in some communities? No, you know, so I think those are, but I don’t think that’s unique to religion, I think that has to do with partnerships in general, are you showing up as an authentic partner? … what’s your purpose? And what is your organization trying to accomplish? Let’s find mutual goals versus like, ‘Hey, how can you help me do my job better,’ which I think public health tends to be because we also feel like we’re helping everybody…” – Participant 8
Focus on building and empowering communities - Avoiding a saviorism mentality - Treating people with respect and equality - Democratizing knowledge - Dealing with relevant contemporary challenges needed by the community organically, enthusiastically, and optimistically - Embracing the expertise of all	“So I think what needs to happen is we need to be more thoughtful about how we meaningful engage with communities, and [engagement] is not all about the program or product or whatever at hand. You build the relationship, and then you bring something in it for them. Right, you also bring them to the table as a decision maker to help design, implement, and evaluate these programs as well. So they are a true stakeholder and decision maker. And you’re not just throwing something at them … they’re actually being engaged and involved in the process. And that’s what’s gonna help build that bridge of trust, is when people feel like they heard your value, they’re seeing their thoughts and inputs matter, because they have been incorporated into whatever you may be working on. One of my biggest pet peeves is ‘don’t ask the person a question if you don’t really want to know the answer.’ If you’re not gonna do anything with it, then just don’t do it. Right? Because it puts a false sense of security on the other person, like, oh, my gosh, they came to me, they asked for my opinion, and I gave them all this information. And then you roll out the program, and they’re looking like, ‘Well, none of what I said was included, so why waste my time.’ And that just puts a bad taste in people’s mouth. So I think just going about it from a more authentic and meaningful way and taking some of that structure and rigor away while you’re trying to build the relationship. And I’m not saying to sacrifice quality by any means. But when you come in the door, you can’t start with all this scientific jargon and research terms. Nobody cares about that. It’s like, what are you here for? What do you want? How can we work together? How is this mutually beneficial for all of us? How is this going to benefit my community? That that’s, that’s where the conversation needs to be? You know, nobody cares about the well thought out and constructed research question. … we’re making sure we’re also presenting information in a way that can be understood and received, and we’re, you know, using plain language principles, for example. So I think that will allow us to be able to build trust within those communities, and then bridge that gap and have a better perception of, you know, what public health does, how do we operate? You know, what value are we bringing, and here’s how we serve communities.” – P5 “I think it’s easy in our public health framework, and this is where I think we’ve really gone off the rails as a field and the last couple of years too, so we think about the poor person, the homeless, or like, I was saying somebody begging on the street or somebody’s ill, but what about somebody who’s an anti vaxxer you know, who was anti COVID, you know, says that whatever we’re doing is bad and wrong. That’s where I don’t think the compassion in our field is there, and I don’t, I’m not criticizing necessarily because it’s very hard to think of somebody who is going against what you’re believing in and you think, ‘Oh, they’re in the image of God like oh, no,’ like you know, I mean, that’s tough. So I think that we in public health tend to have the more, what’s the word I want to say, instinct of you know, saviorism, right. We’re here to help the poor person or the honorable person. But I think in our, in our own heart, you have to think about this more, of somebody who disagrees with me. Why do they disagree with me rather than they’re wrong and I’m right, which tends to be where we’re at … But in general, I think public health approaches things in a way that we’re right. And we’re helping, we’re here to help you with the savior type approach. So being like, hey, what do you all need? And how can we help you? And then let’s think about this. Versus we know what’s right.” – Participant 8
Building comfort with engaging religious and spiritual communities may first require specific tasks from public health specialists - Acknowledging spirit and/or the divine - Embracing faith-based language - Embracing complexity in measurement	“…I went to South Sudan, and I’m, I’m with the church, and I’m going to do this health program. And I tell the people in advance [that] I’m coming to do a health program, and I show up at the village, and like, no one’s, there’s like 10 people there. And I’ve started talking about mosquito nets and pit latrines and stuff, and people out there getting sleepy … or they couldn’t care less, right, despite the fact that Malaria is endemic. And despite the fact that people are dying from dysentery, you know, every other day… I couldn’t get them to listen. And then when I go to town, and I say, ‘Hey, we’re gonna do a revival’ or something, then everyone, the whole village shows up, right? And they’re all there. They’re playing music, and like, they got all these little kids running around. … I’m gonna teach them help through the Scripture lessons, through the Bible lessons. … And so, I go there and I say, ‘Okay. When Mary, gave birth to Jesus, The Bible says that she wrapped him in swaddling clothes.’ And they said, ‘Yeah,’ ‘Well, what? Why did she wrap him in swaddling clothes? Right? Why didn’t she just lay on, and, you know, in English, we say laying them in the manger. In other places, they say lay him in the feeding trough, which was what a manger is.’ … And so, you know, the answer is, well, you know, maybe it was dirty, or maybe the insects want to bite them or something she didn’t want them to. They didn’t. Maybe it was cold. She wanted them to stay warm. And all those are great answers, right? And so, but in a nutshell, you’re saying that you’re trying to protect him from the environment, whatever harms the environment has, wrapping in this will enclose [and] protect them from the environmental harms. If Mary gave birth to Jesus in Sudan, South Sudan, and there’s like, this pesky mosquito that keeps biting everybody, and making everybody sick, do you think Mary’s gonna protect them from the environment? Or Mary’s gonna lay on there? And then we get a bit? And so they said, I think Mary’s gonna protect them. Right? So, yeah, that’s right. But so I was talking to you, we had this thing called the mosquito net, and you’ve been using it to go fishing and you’ve been using it to like, even throwing it away and like rejecting it. But you know, you can put it in, keep your baby from getting sick, and be like Mary. And, you know, you know, whole villages come back a month later, and everyone has a mosquito net … And I was I was so impressed by it. Because it was intertwined. Like the message, it was intertwined in the way that they couldn’t. You couldn’t just break it. It wasn’t science and it wasn’t religion. It was like, it was a package. And so that package, you know, moved them in both directions.” – Participant 4 “And increasingly over the years, I’ve noted that we’ve discounted the role of religion and spirituality in population science, completely discounted it. And when we do account for it, we only look at the extreme versions of religion. So we tend to look at religious extremism, whether it’s an evangelical Christian or Hindu, right wing Hinduism, or Islam, whatever the extreme is, we tend to say ‘see they’re not science, or they don’t believe in science.’ Well, yeah, but that’s not the only space to understand religion. I’ve also been dabbling with something that [is] the sociological constructs of understanding religiosity. And there’s three variables that I’m super interested in that scientists have tried to explain, for example, the decrease in religiosity in the United States, and they look at three main variables, and that’s belief, belonging, and behavior. And what I’ve noticed in my dabble, because I’m not a I’m not a religious scholar, but nor am I a sociologist of religion, but what I’ve seen in the studies, and I’m gonna tell you why I’m linking this to help in a second. What I’ve seen is that scholars, mostly statisticians, right, social scientists, look at the three variables separately. And they’ll make a determination based on one set of variables. So like, they’ll look at belonging, as, do you belong to a church? Yes or no? Do you belong to a temple? Yes, or no? Kind of like this very easy to analyze and get robust models from, but doesn’t really address religion as a whole, and how that impacts health and well being. … I’ve noticed these studies that show a decrease in religiosity based on the fact that people have either withdrawn from a religious organization, organized religion. So if you look at that, you see rates of religion declining in the United States, for example. But if you look at just belief, we also need to unpack what does that mean to believe in something? And this goes back to the earlier question of science, I mean, the earlier concept of science and religion being systems of knowledge, there are some people, my students included, who will identify with science as though it’s their religion. So I have these little get to know you quizzes, like in the beginning of class, just so that I get to know some of the statistics of my classes, and I asked a question about their religious beliefs. Increasingly, over the years, I’ve noticed, anecdotally, my students are saying, I don’t believe in religion. But then, when we have a conversation about do you believe in a greater entity, do you believe in something, you don’t have to call it God, I also don’t believe God is a white man in the sky throwing lightning bolts at us, like you don’t have to believe in that construct of a Judeo Christian God like Santa Claus, but do you believe in something greater than yourself? ‘Yeah, I do. I just don’t like the way that I see it play out in society.’ So it’s like that construct of belief needs to be unpacked, the construct of belonging also needs to be expanded, what is it? You don’t have to be belong to a temple, a church, to feel like you belong in a community. None of these models really help us understand the Baha’i faith, actually, because the behavior is so different. It doesn’t have a church, right? So if you answer that question as a Baha’i, well, no, I’m not part of a church. But it doesn’t give you the option of community.” – Participant 9

## Discussion

Public health professionals frequently encounter religious beliefs and practices in their work, but few studies have explored how they perceive religion, navigate these encounters, or how they integrate religious considerations into their engagement strategies. In this study, a qualitative descriptive methodology using interviews served to begin filling this gap by exploring several important findings on how public health practitioners define and understand religion and public health, as well as conceptualize the relationship between the two systems of knowledge and practice.

In this study, practitioners reported a wide range of experiences, reflecting the complex ways in which understanding of religion and spirituality evolve over time based on one’s lived experience, as well as how these experiences and perspectives intersect with practitioners’ understanding of public health objectives, and how religion can help shape public health responses, particularly during health emergencies like the COVID-19 pandemic. Findings underscored that while some religious communities adhere to public health recommendations, others may hold beliefs that challenge adherence, making engagement between public health and religious communities an opportunity and a challenge to navigate. In this regard, this study highlights the importance of culturally and religiously competent public health strategies to enhance trust and meaningful collaboration.

In recent years, the relationship between religion, and public health has been gaining increased recognition as an essential factor for positively influencing health outcomes, ranging from mental well-being to promoting physical activity [26]. Faith-based interventions have also shown promising results in addressing health disparities, especially in communities that experience limited access to formal healthcare [10, 17]. As religion often acts as a bridge between communities and healthcare, the need to ensure that communication between religious and public health communities promotes safety, respect, and empowerment is critical [27].

In the present study, both alignment and tension between religion and public health communities are observed. Findings suggest that fundamentalist perspectives, in particular, can sometimes lead to resistance against certain health interventions, such as vaccination campaigns, as previously noted in other studies [21]. Concurrently, our findings align with Kemp’s insights on how faith-based partnerships enhanced COVID-19 compliance [28]. Also, in this study, we note instances of public health enforcing their beliefs on religious communities, suggesting that both communities can be prone to fundamentalist tendencies. To counteract this, a critical need to adopt principles of community-based research is essential. For example, humility, as a quality in public health engagement, was noted as essential for practitioners as it fosters genuine relationship-building with religious communities. Training programs in public health could benefit from incorporating humility, cultural sensitivity, and cultural safety as foundational elements in community health education [26].

Our research reinforces the need to foster dialogue between science and religion, with the goal of bridging differences and finding common ground. The past few years of the COVID-19 pandemic have also exposed how the broader discourse on science and religion is not an abstract ideological debate, but an issue with real-world consequences. Recent research has underscored the role of religion in shaping health behaviors during global crises [27–29]. Vaccinations and compliance with behavioral measures to reduce the spread of COVID-19 were met with resistance from some religious communities, while being supported by others [21, 30, 31]. Religious leaders and institutions often become important gatekeepers of health information and can either reinforce or challenge public health directives. Studies from the pandemic have shown that in some communities, religious practices were adjusted to align with health guidelines, while in others, religious beliefs complicated compliance with public health measures [30]. Religious institutions also provided essential communication platforms, disseminated health messages, and supported adherence to health directives, especially in marginalized communities [12, 28]. For many participants in the present study, both science and religion serve shared goals of promoting well-being and community cohesion, suggesting that these fields are not inherently oppositional but complementary [32]. However, political or societal discourses often polarize science and religion, which can complicate their integration in public health.

Our findings suggest that encouraging open and respectful dialogue that explores the synergies between scientific and religious knowledge is vital for advancing public health policies that resonate with diverse populations. This balanced approach could help alleviate tensions, fostering trust and collaboration between religious communities and health practitioners. Examples of support for this approach can be found in the literature. Thompkins and colleagues, for example, described the Project Trust Model – a collaborative model encouraging an ecological approach for incorporating African American pastors into the fight against COVID-19 and public health services [33]. Overwhelmingly, the model has proven a success by ensuring community members feel heard and that their trust in public health authorities is supported by their Church. As DeBoer also observed, early in the COVID pandemic, the State of Alabama adopted an innovative approach of constructive engagement with religious leaders and communities by forming a task force of religious leaders, medical personnel, and the former head of the Alabama Department of Public Health to co-develop guidelines for public health measures and timely reopening [34]. These findings and creative arrangements demonstrate the need for public health practitioners to understand the religious dynamics of the populations they serve, to enhance health literacy, and build trust with religious communities [31]. This understanding can also lead to more effective health campaigns and policy implementations that are respectful of religious practices while promoting public health objectives.

Additionally, contemporary research also outlines what a meaningful relationship between science and religion could look like. For example, A comprehensive collection by Doug Oman entitled, “*Why Religion and Spirituality Matter for Public Health?*” summarizes over 3000 articles and attests to the need for and practical steps involved with greater collaborations between science, public health, and religion [14]. Another example which seeks to advance understanding on how the investigation of truth and the advancement of civilization can be achieved through integrating science and religion comes from Paul Lample’s “*In Pursuit of Harmony between Science and Religion*” [35]. There are also national organizations, such as the American Association for the Advancement of Science’s Dialogue on Science, Ethics, and Religion among others, that aim to facilitate broader communication between scientific and religious communities [36]. Furthermore, Long et al.’s discussion on person-centered, evidence-based, spiritual care also provides a useful framework for public health practitioners who engage with religiously diverse populations [4]. By outlining how health systems can integrate spiritual care services, such as chaplaincy and spiritual assessments, the article underscores how public health can become more holistic. Furthermore, the recommendation for training healthcare providers in spiritual care speaks to the need for cultural and religious competence, particularly in handling sensitive issues related to religious beliefs. Importantly, this capacity building to more meaningfully engage religious communities can help public health practitioners gain the ability to better serve all communities.

While our study offers important insights, it also has limitations. Firstly, the work conducted follows a qualitative descriptive methodology using interviews. While this is excellent for describing and initially exploring perspectives of participants, which is the purpose of this study, this methodology is not well suited to explain concepts comprehensively (e.g., why a particular perspective might be held). Future research should focus on using more robust qualitative methodologies such as case studies, grounded theory, or ethnography, to more thoroughly understand why certain perspectives exist, the factors and context within which positive and negative collaboration emerge, and what harmonious collaboration between religious and public health communities could look like. Furthermore, our sample size, though diverse, may not capture the full range of perspectives across different religious backgrounds. Importantly, we also only explored the perspectives of public health practitioners. Future research should aim to explore the perspectives of religious leaders to more fully understand the phenomenon of collaboration through their experiences. Additionally, the qualitative nature of our interviews, and particularly, the framework analysis approach taken, offers exploratory insights but may limit generalizability. Future research should consider larger, quantitative studies to examine these dynamics across broader populations. Moreover, longitudinal studies could provide a deeper understanding of how relationships between religious communities and public health evolve, especially during prolonged public health crises. Future research should also address methodological challenges in measuring religious attitudes and beliefs to refine public health approaches that respect diverse spiritual frameworks.

## Conclusion

The findings from this research underscore the need for a more integrated approach between religion and public health. As global public health challenges continue to arise, fostering collaboration with religious communities will be essential. The COVID-19 pandemic highlighted both the strengths and limitations of current health systems in engaging with religious groups effectively. Building stronger, mutually respectful partnerships between public health and religious institutions can prepare societies for future health emergencies by ensuring that public health interventions are both scientifically sound and culturally accepted. This integration could contribute to more resilient public health systems globally.

## Supplementary Information

Supplementary Material 1

Supplementary Material 2

Supplementary Material 3

## Data Availability

The datasets generated and/or analysed during the current study are not publicly available because of privacy concerns; participants are potentially identifiable due to the small sample and overall population size, and because of the qualitative nature of much of the data. The datasets are potentially available from the corresponding author on reasonable request.

## Abbreviation

COREQ: COnsolidated criteria for REporting Qualitative research
