# Supplementary figures and images for: Religion and public health: conceptualization and collaboration from a public health perspective
Source: BMC Public Health. 2026 Jan 29;26:701. doi: 10.1186/s12889-026-26411-7 (PMC12924531; doi:10.1186/s12889-026-26411-7)

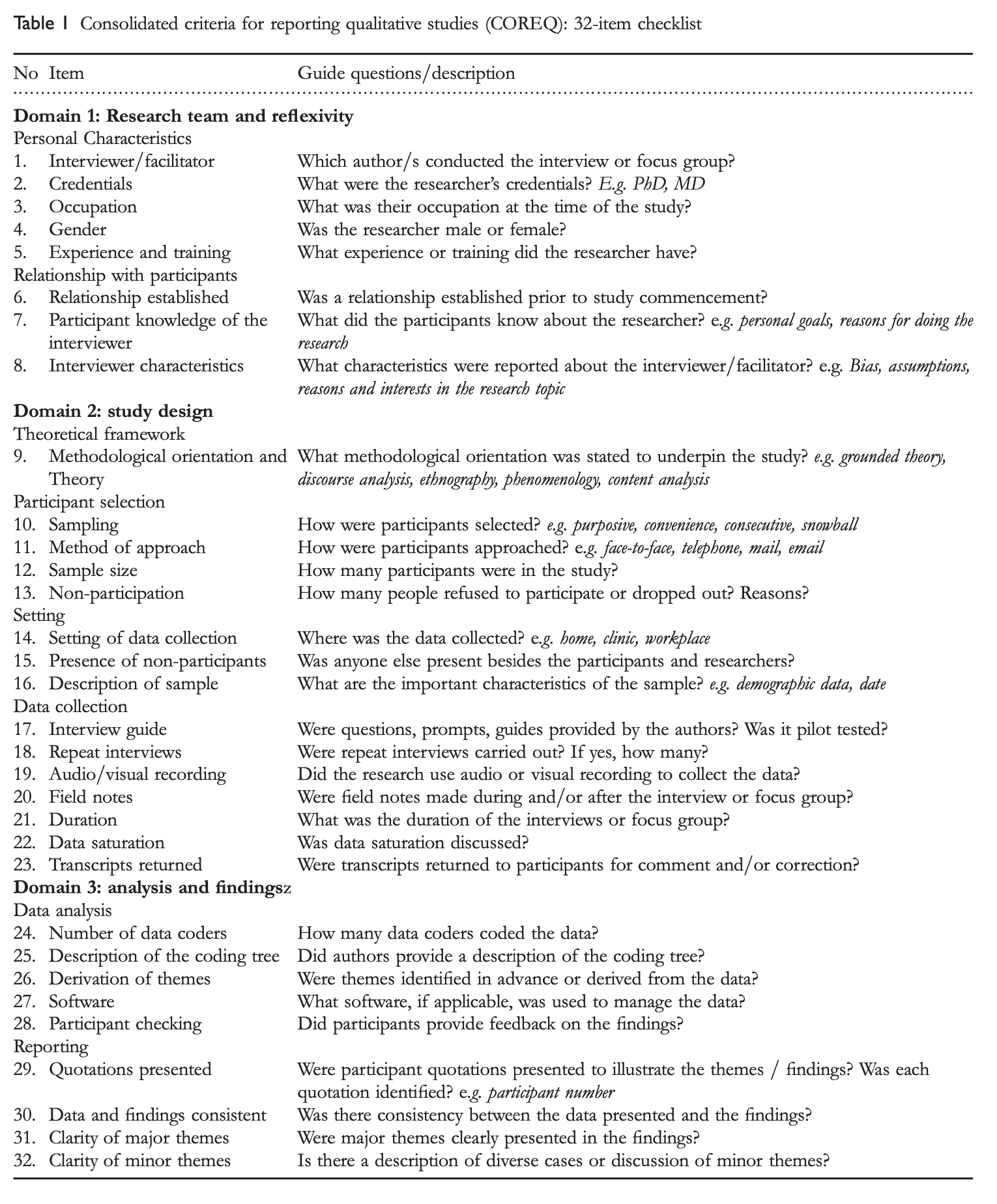

Supplement: Supplementary file 1 — Supplementary Material 1 [file 12889_2026_26411_MOESM1_ESM.docx]
