# Supplementary material for: Religion and public health: conceptualization and collaboration from a public health perspective
Source: BMC Public Health. 2026 Jan 29;26:701. doi: 10.1186/s12889-026-26411-7 (PMC12924531; doi:10.1186/s12889-026-26411-7)
Supplement: Supplementary file 2 — Supplementary Material 2 [file 12889_2026_26411_MOESM2_ESM.docx]

Appendix 2: Interview questions:

*Background/Introduction:*

< Interviewer note: Introduce yourself and describes the project: The purpose of this project is to develop a better understanding of the engagement between public health and religious communities during the COVID-19 pandemic, and more broadly. We are conducting a series of interviews with leaders from the public health community and a range of religious communities to explore the engagement between the two groups.>

- Can you tell us briefly about your professional background and work experience?
  - What was your professional role during the COVID-19 pandemic?
- Can you share with us your personal Faith orientation or background?

*Defining the Public health and Religion*

<Interviewer note: Flip the order of these questions based on who is being interviewed (e.g., public health practitioner could be asked their understanding of public health before talking about religion>

- How would you define or understand the field of public health?
  - What are its main goals and aspirations from your view or experience?
  - What are the main assumptions about human nature underpinning public health?
- How would you define religion?
  - What are its main goals and aspirations from your view or experience?
  - What are the main assumptions about human nature in religion?
  - In your opinion, what is the relationship between religion and spirituality?
- From your experience, how would you say people from various religious backgrounds or religious communities view the field of public health?
  - What about their view on the scientific community in general?

*Drawing from Practical Experiences*

- From your experience, how would you say public health practitioners view religion or people of faith?
- What do you see as the current relationship between religion or faith communities and public health?
  - Are there processes that you feel can help facilitate this relationship? Any examples of dynamics you’ve seen in practice?
  - Are there barriers you feel that can hinder this relationship? Any examples of dynamics you’ve seen in practice?
  - How do these barriers and facilitators impact the day-to-day health and wellness of individuals within specific populations you may have worked with or been a part of?
  - From your view, do these barriers or facilitators vary for minority or majority populations across a community? Or across the whole country? *(In other words, how might these relationships or lack thereof impact different ethnic or religious populations in different ways?)*
- During the COVID-19 pandemic, did you witness any times when religion and public health interacted in some way?
  - Can you share any stories or instances you were a part of or observed?
  - Where there times when this interaction worked well or benefited communities?
  - Where there times when this interaction didn’t work well or harmed communities?
- Beyond the COVID-19 pandemic, have you observed any times when people of faith and public health practitioners interacted or worked together in some ways?
  - What do you think this relationship between religion and public health could or should look like?
  - What are some benefits or drawbacks of an improved working relationship between religious communities and the field of public health?
  - What steps may be needed to improve a relationship between religion and public health?
- What have you learned from this experience?

*Closing and wrap up*

- Anything else you would like to share about the working relationship between religion and public health?
